# The Outcomes of Cardiac Surgery in Children With DiGeorge Syndrome in a Single Center Experience: A Retrospective Cohort Study

**DOI:** 10.7759/cureus.55186

**Published:** 2024-02-28

**Authors:** Tala M AlAshgar, Norah H AlDawsari, Nasreen Y AlSanea, Noura A AlSalamah, Nada S AlSugair, Husam I Ardah, Mohamed S Kabbani

**Affiliations:** 1 Medicine and Surgery, King Saud Bin Abdulaziz University for Health Sciences College of Medicine, Riyadh, SAU; 2 Biostatistics and Epidemiology, King Abdullah International Medical Research Center, Riyadh, SAU; 3 Pediatric Critical Care, Department of Cardiac Sciences, Ministry of the National Guard Health Affairs, King Abdullah International Medical Research Center, King Saud Bin Abdulaziz University for Health Sciences, Riyadh, SAU

**Keywords:** digeorge syndrome, congenital heart diseases, 22q11.1 deletion syndrome, post operative outcome, pediatric cardiac surgery

## Abstract

Background

DiGeorge syndrome, a common genetic microdeletion syndrome, is associated with multiple congenital anomalies, including congenital cardiac diseases. This study aims to identify the short and midterm outcomes of cardiac surgery performed on children with DiGeorge syndrome.

Methods

A retrospective cohort study was conducted between the period of 2018-2022, which included children divided into two groups with a 1:2 ratio. Group one included DiGeorge syndrome patients who were diagnosed using fluorescence in situ hybridization (FISH). Group two included the control group of patients who were clear of genetic syndromes. The two groups were matched based on similar cardiac surgery, age of surgery, and Risk Adjustment in Congenital Heart Surgery (RACHS-1) score. The two groups were compared based on the demographical data and postoperative complications.

Results

The study consisted of 81 children; 27 were DiGeorge syndrome patients, and 54 were in the control group. DiGeorge syndrome patients showed an increase in mechanical ventilation duration (p=0.0047), intensive care unit (ICU) length of stay (p=0.0012), and hospital length of stay (p=0.0391). Moreover, they showed an increased risk for bacteremia (p=0.0414), ventilator-associated pneumonia (VAP; p=0.0036), urinary tract infections (UTI; p=0.0064), and surgical site infection (SSI; p≤0.0001). They were also more susceptible to postoperative seizures (p=0.0049). Furthermore, patients with DiGeorge syndrome had a higher prevalence of congenital renal anomalies. However, there was no mortality in either group.

Conclusion

This study shows a variability in the postoperative outcomes between the two groups. The study demonstrates that patients with DiGeorge syndrome have higher risks of infections and longer hospital stay during the postoperative period. Further research with a larger sample is needed to confirm our findings.

## Introduction

DiGeorge syndrome (DGS) is one of the most prevalent genetic microdeletion syndromes that occurs in approximately 1:3000-6000 births [1-2]. It is caused by a proximal 1.5 Mb deletion of the long arm of chromosome 22 [3]. Therefore, it is diagnosed using a targeted deletion analysis, such as fluorescence in situ hybridization (FISH) analysis [4]. The main mutated gene in DiGeorge syndrome is T-box transcription factor 1 (TBX1), which is responsible for provoking the features of DiGeorge syndrome, like hypoparathyroidism, thymic hypoplasia, abnormal facial features, and congenital heart defects [5]. 

The major conotruncal heart defects associated with DiGeorge syndrome include tetralogy of Fallot, pulmonary atresia with ventricular septal defect, truncus arteriosus, interrupted aortic arch type B, isolated anomalies of the aortic arch, and transposition of great arteries [6]. There is no definitive cure for DiGeorge syndrome. However, children with DiGeorge syndrome who manifest with heart defects are usually corrected by surgeries [7]. There are multiple surgical procedures that are frequently operated on these patients to correct these critical congenital heart defects, which may include arch surgery, tetralogy of Fallot repair, systemic-to-pulmonary shunts, valvular operations, and unifocalization procedures [8]. Without cardiac surgery, children can only live for two or three years. Moreover, over the patient's lifetime, many other non-cardiac surgeries may be necessary due to the other manifestations of DiGeorge syndrome. Furthermore, an article issued in 2017 by Alsoufi et al. compared the effects of cardiac surgery identified on neonates with DiGeorge syndrome and neonates with no genetic syndromes. Subsequently, DiGeorge syndrome patients showed increased complications and comorbidities compared to the patients with no genetic syndromes [9]. During the past years, there has been a paucity of data about the outcomes of DiGeorge syndrome patients undergoing cardiac surgery. Therefore, we highlight the necessity for a research paper that will analyze these effects in the current era to ensure the patients' lifetime. In our research, we aim to identify the short-term and midterm outcomes of cardiac surgery performed on children with DiGeorge syndrome while comparing them with children with no genetic syndromes.

## Materials and methods

This is a retrospective cohort study that was conducted in the pediatric cardiac intensive care unit (ICU) in a tertiary center. The center receives an average admission of 500 pediatric cardiac patients every year, with annual cardiac surgeries reaching approximately 350 patients per year. The study's inclusion criteria consisted of children between birth and 14 years of age. These children were confirmed by FISH analysis that they have DiGeorge syndrome and congenital cardiac diseases which they underwent cardiac surgery for. These patients were compared to a control group, a matching group with no genetic syndromes, who had comparable cardiac diseases, age of surgery (in months), and Risk Adjustment in Congenital Heart Surgery (RACHS-1) score. RACHS-1 score is a score that anticipates the risk of mortality in patients under the age of 18 after cardiac surgery [10]. The data was obtained from digital medical records after obtaining approval from the institutional review board (number 1646/22).

The patient's data included independent variables and outcome variables. The independent variables were age, gender, type of cardiac disease, type of surgery, and associated somatic diseases. On the other hand, the outcome variables were the short-term and midterm postoperative outcomes. The short-term outcomes were defined as the survival and the events that occurred less than a year after the surgery. This included the requirement for extracorporeal membrane oxygenation (ECMO) support, postoperative mechanical ventilation duration, ICU length of stay, hospital length of stay, infections including bacteremia, ventilator-associated pneumonia (VAP), urinary tract infection (UTI), and surgical site infection (SSI), and surgical mortality (defined as death that occurred within 28 days after surgery). In contrast, the midterm outcomes were the events that occurred within one to five years, including the duration of the patient's follow-up and mortality. 

Statistical analysis was conducted using SAS 9.4 (SAS Institute Inc., Cary, USA). The means and proportions of the study participants were calculated to characterize the study participants overall and in groups (DiGeorge syndrome, no DiGeorge syndrome control). The two groups were compared using the Chi-square or Fisher exact test for categorical factors and the t-test or Kruskal Wallis Test for continuous variables as appropriate. The level of significance was declared at α=0.05 and a confidence interval of 95%. The effect sizes were reported using Cohen's D and its confidence interval for continuous variables and relative risk with its confidence interval for binary variables.

## Results

As Table 1 indicates, a total of 27 DiGeorge syndrome patients underwent cardiac surgery, while the control group consisted of 54 patients. The average age at the time of the surgery for DiGeorge syndrome patients and control group patients was 6.7±14.8 months and 9.1±21.6 months (p=0.4258), respectively. Preoperatively, DiGeorge syndrome patients exhibited a significantly lower T cell count, 2.9±5.7^9^/L, compared to the control group, 11.5±4.0^9^/L (p≤0.0001). Eight DiGeorge syndrome patients were confirmed to have an absent thymus during surgery. In addition, DiGeorge syndrome patients had a lower total calcium count, 1.9±0.3 mmol/L, in contrast to the control group, 2.2±0.7 mmol/L (p=0.0149). Furthermore, in the DiGeorge syndrome group, nine patients (33.3%) had renal anomalies. On the other hand, only five (9.3%) patients in the control group had renal anomalies (p=0.0169). 

**Table 1:** Demographic data of DiGeorge syndrome and control groups RACHS-1 score - Risk Adjustment for Congenital Heart Surgery score

Variables	DiGeorge syndrome group (n, %; mean±SD)	Control group (n, %; mean±SD)	p-value
Male	14 (51.9%)	28 (51.9%)	0.9999
Female	13 (48.1%)	26 (48.1%)	0.9999
Age of surgery (months)	6.7±14.8	9.1±21.6	0.4258
RACHS-1 score	3.1±0.92	2.8±0.9	0.3550
Preoperative T cell count (10^9^/L)	2.9±5.7	11.5±4.0	<0.0001
Preoperative total calcium count (mmol/L)	1.9±0.3	2.2±0.7	0.0149
Renal anomalies	9 (33.3%)	5 (9.3%)	0.0169

The most common cardiac diseases seen in DiGeorge syndrome patients were truncus arteriosus and ventricular septal defect, as seen in Table 2. The second most prevalent type observed was tetralogy of Fallot. Interrupted aortic arch and other types of cardiac diseases were among the least common conditions, as they were identified in only two patients each. 

**Table 2:** Cardiac diseases details in DiGeorge syndrome and control groups Others include atrial septal defect, patent ductus arteriosus, and aortic stenosis.

Cardiac disease	DiGeorge syndrome group (n=27)	Control group (n=54)	p-value
Truncus arteriosus	9 (33.3%)	18 (33.3%)	0.8787
Ventricular septal defect	9 (33.3%)	18 (33.3%)	0.2606
Tetralogy of Fallot	5 (18.5%)	10 (18.5%)	0.9414
Interrupted aortic arch	2 (7.4%)	4 (7.4%)	0.643
Others	2 (7.4%)	4 (7.4%)	0.0685

Postoperatively, DiGeorge syndrome patients presented with more cases of bacteremia (n=10, p=0.0414), VAP (n=12, p=0.0036), UTI (n=18, p=0.0064), SSI (n=22, p≤0.001), and seizures (n=6, p=0.0049) than the control group patients. Furthermore, DiGeorge syndrome patients required more time for mechanical ventilation, 42.6±80.6 days, compared to control group patients, 9.3±10.7 days (p=0.0047). DiGeorge syndrome patients have also spent more time in the ICU, 30.3±32.9 days, as opposed to the control group patients, 11.9±8.3 days (p=0.0012). Moreover, DiGeorge syndrome patients required more hospital length of stay, 76.9±114.1 days, than the control group patients, 33.6±22.5 days (p=0.0391; see Table 3). However, there was no difference in the mortality between the two groups for both short-term and midterm outcomes, with 100% survival in both groups.

**Table 3:** Postoperative outcomes variables in DiGeorge syndrome and control groups *Cohn's D effect and its confidence interval for continuous variables. **Relative risk and its confidence interval for binary variables. VAP - ventilator-associated pneumonia; UTI - urinary tract infection; SSI - surgical site infection; ICU - intensive care unit; ECMO - extracorporeal membrane oxygenation

Postoperative complications	DiGeorge syndrome group (n, %; mean±SD)	Control group (n, %; mean±SD)	Effect size (95% confidence interval)	p-value
Bacteremia	10 (37%)	9 (16.7%)	2.2222 (1.0258, 4.8140)**	0.0414
VAP	12 (44.4%)	8 (14.8%)	0.9615 (0.8538, 1.0829)**	0.0036
UTI	18 (66.7%)	16 (29.6%)	0.7826 (0.5862, 1.0448)**	0.0064
SSI	22 (81.5%)	0	0.5926 (0.4334, 0.8102)**	<0.0001
Seizures	6 (22%)	1 (1.9%)	0.7925 (0.6456, 0.9727)**	0.0049
Mechanical ventilation duration (days)	42.6±80.6	9.3±10.7	0.71647 (0.24119, 1.19176)*	0.0047
ICU length of stay (days)	30.3±32.9	11.9±8.3	0.92873 (0.44460, 1.41286)*	0.0012
Hospital length of stay (days)	76.9±114.1	33.6±22.5	0.64475 (0.17197, 1.11753)*	0.0391
Acute renal injury	1 (3.7%)	7 (12.9%)	1.1274 (0.9817, 1.2946)**	0.9602
Pacemaker need	3 (11.1%)	4 (7.4%)	0.9600 (0.8236, 1.1190)**	0.6868
ECMO	1 (3.7%)	0	0.9630 (0.8943, 1.0369)**	0.3333
Chylothorax	0	7 (12.9%)	1.1489 (1.0366, 1.2735)**	0.1636

## Discussion

In our study, it has been revealed that ventricular septal defect and truncus arteriosus are the most commonly detected cardiac diseases in DiGeorge syndrome patients. Tetralogy of Fallot was identified as the second most common type. These findings contrast with previous studies, which suggest that tetralogy of Fallot is the most common type of cardiac disease among DiGeorge syndrome patients [6-11]. 

The postoperative course for DiGeorge syndrome patients was different from the control group patients in terms of infection rates and overall hospital length of stay. DiGeorge syndrome patients had a higher risk of developing postoperative infections. They are known to be immunodeficient due to their congenital thymic hypoplasia or absent thymus; thus, they had a deficiency of preoperative T-cell count. Among our patients who had DiGeorge syndrome, 10 (37%) had severe immunodeficiency with a lymphocyte count of less than 1000 cells/mm^3^. This immunodeficiency could have predisposed DiGeorge syndrome to a higher risk of developing infections. In our research, an increased number of DiGeorge syndrome patients have acquired bacteremia (37%), VAP (44.4%), UTI (66.7%), and SSI (81.5%) after undergoing cardiac surgery. While in the control group, only 16.7% have developed bacteremia, 14.8% VAP, 29.6% UTI, and no SSI. The elevated incidence of UTIs in DiGeorge syndrome patients may also be attributed to their higher prevalence of congenital renal anomalies. Nine (33.3%) DiGeorge syndrome patients exhibited renal anomalies, whereas only five (9.3%) patients in the control group did. Similarly, another study has found that 30% of DiGeorge syndrome patients present with congenital renal and urinary tract anomalies [12]. Moreover, all DiGeorge Syndrome patients with SSI were managed with antibiotics and daily dressing changes with wound care without the need for surgical debridement. Furthermore, a different study has observed a higher number of cases of bacteremia, UTI, and SSI in DiGeorge syndrome patients, which is comparable to our study [13].

DiGeorge syndrome patients required longer postoperative mechanical ventilation duration, ICU length of stay, and hospital length of stay, which could be attributed to the higher incidence of recurrent nosocomial infections. Similarly, another article published in 2014 had comparable findings that DiGeorge syndrome patients required more hospital and ICU length of stay and longer intubation period [14]. On the other hand, a recently published study in 2012 revealed that there was no significant difference in mechanical ventilation duration, hospital length of stay, and ICU length of stay between DiGeorge syndrome patients and control group patients. However, DiGeorge syndrome patients who have undergone cardiac surgery for tetralogy of Fallot have had a longer duration of hospital and ICU stays [13]. There are other risk factors not related to DiGeorge syndrome, which could contribute to the increased length of stay in DiGeorge syndrome patients, such as the presence of restrictive right ventricle physiology in tetralogy of Fallot or different surgical skills and variable institutional experience [15]. 

The DiGeorge syndrome group presented with a notably decreased number of preoperative calcium levels due to congenital hypoparathyroidism, which could be the possible etiology for the increased rate of developing seizures post-cardiac surgery. Fifty percent of the seizures observed in DiGeorge syndrome patients were of the focal type. Similarly, another study found a higher incidence of seizures in DiGeorge syndrome patients in both preoperative and postoperative periods in comparison to a control group free of genetic syndrome. However, the higher risk of seizures in DiGeorge syndrome patients was not linked to the low calcium levels in the postoperative period [16]. Furthermore, only one DiGeorge syndrome patient required ECMO after undergoing tetralogy of Fallot repair, and it was not statistically significant compared to the control group. Congenital anomalies such as cleft lip, cleft palate, and craniofacial abnormalities were only present in the DiGeorge syndrome group. 

In terms of postoperative mortality, Alsoufi et al.'s article showed that mortality rates were higher in DiGeorge syndrome patients, while the two groups in our research had all survived [9]. Nonetheless, a study by Yeoh et al. published in 2014 showed that patients with DiGeorge syndrome who have had cardiac surgery had a 100% survival rate during the hospital and 30 days after discharge, which goes in parallel with our research [8].

Our study's strength lies in its retrospective cohort design, which enables the evaluation of multiple outcomes coming from a single exposure and the assessment of its impacts. Furthermore, evaluating the postoperative outcomes in a subgroup of children with a unique syndrome requiring early-stage cardiac surgery contributes additional information to the existing literature on the outcomes. The limitation of this retrospective study was selection bias, as shown in the sample size and the study technique. A larger number of DiGeorge syndrome patients and control group patients is required to acquire more accurate results, as this paper had a small sample size. In addition, a non-randomized matching technique was used for the two groups based on specific factors. While we believe these limitations have not affected the primary outcome of the study, future research could aim to include a larger sample size involving multiple centers and randomized techniques.

## Conclusions

In conclusion, this study shows a significant variability between the DiGeorge syndrome group and the control group in terms of postoperative comorbidities. The mechanical ventilation duration, ICU length of stay, hospital length of stay, infection rates, and seizures were higher in the DiGeorge syndrome group. As such, the importance of the study is that it will inform medical practitioners about the postoperative effects that DiGeorge syndrome patients are exposed to following correction of their cardiac lesions. 

## References

[1] Kraus C, Vanicek T, Weidenauer A (2018). DiGeorge syndrome: relevance of psychiatric symptoms in undiagnosed adult patients. Wien Klin Wochenschr.

[2] Cortés-Martín J, Peñuela NL, Sánchez-García JC, Montiel-Troya M, Díaz-Rodríguez L, Rodríguez-Blanque R (2022). Deletion syndrome 22q11.2: a systematic review. Children.

[3] Funato N (2022). Craniofacial phenotypes and genetics of DiGeorge syndrome. J Dev Biol.

[4] Bahamat AA, Assidi M, Lary SA (2018). Use of array comparative genomic hybridization for the diagnosis of DiGeorge syndrome in Saudi Arabian population. Cytogenet Genome Res.

[5] Kobrynski LJ, Sullivan KE (2007). Velocardiofacial syndrome, DiGeorge syndrome: the chromosome 22q11.2 deletion syndromes. Lancet.

[6] Unolt M, Versacci P, Anaclerio S (2018). Congenital heart diseases and cardiovascular abnormalities in 22q11.2 deletion syndrome: From well-established knowledge to new frontiers. Am J Med Genet A.

[7] McDonald-McGinn DM, Sullivan KE, Marino B (2015). 22q11.2 deletion syndrome. Nat Rev Dis Primers.

[8] Yeoh TY, Scavonetto F, Hamlin RJ, Burkhart HM, Sprung J, Weingarten TN (2014). Perioperative management of patients with DiGeorge syndrome undergoing cardiac surgery. J Cardiothorac Vasc Anesth.

[9] Alsoufi B, McCracken C, Shashidharan S, Deshpande S, Kanter K, Kogon B (2017). The impact of 22q11.2 deletion syndrome on surgical repair outcomes of conotruncal cardiac anomalies. Ann Thorac Surg.

[10] Jacobs JP, Jacobs ML, Lacour-Gayet FG (2009). Stratification of complexity improves the utility and accuracy of outcomes analysis in a Multi-Institutional Congenital Heart Surgery Database: Application of the Risk Adjustment in Congenital Heart Surgery (RACHS-1) and Aristotle Systems in the Society of Thoracic Surgeons (STS) Congenital Heart Surgery Database. Pediatr Cardiol.

[11] Goldmuntz E (2020). 22q11.2 deletion syndrome and congenital heart disease. Am J Med Genet C Semin Med Genet.

[12] Lopez-Rivera E, Liu YP, Verbitsky M (2017). Genetic drivers of kidney defects in the DiGeorge syndrome. N Engl J Med.

[13] McDonald R, Dodgen A, Goyal S (2013). Impact of 22q11.2 deletion on the postoperative course of children after cardiac surgery. Pediatr Cardiol.

[14] O'Byrne ML, Yang W, Mercer-Rosa L (2014). 22q11.2 Deletion syndrome is associated with increased perioperative events and more complicated postoperative course in infants undergoing infant operative correction of truncus arteriosus communis or interrupted aortic arch. J Thorac Cardiovasc Surg.

[15] Sandeep B, Huang X, Xu F, Su P, Wang T, Sun X (2019). Etiology of right ventricular restrictive physiology early after repair of tetralogy of Fallot in pediatric patients. J Cardiothorac Surg.

[16] Bonilla FA (2004). A cohort study of neurodevelopmental outcome in children with digeorge syndrome following cardiac surgery. Pediatrics.

